# Deciphering the complex life cycle and partial migration of an ecological engineer and critical Neotropical fishery species, *Prochilodus costatus*

**DOI:** 10.1111/jfb.70297

**Published:** 2025-12-05

**Authors:** Alexandre Peressin, João de Magalhães Lopes, Francisco Ricardo Andrade Neto, Gilmara Junqueira Machado, Carlos Bernardo Mascarenhas Alves, Luis David Solis Murgas, Paulo Santos Pompeu

**Affiliations:** ^1^ Departamento de Ecologia e Conservação Universidade Federal de Lavras, campus Universitário Lavras Brazil; ^2^ Centro de Ciências da Natureza, Universidade Federal de São Carlos (UFSCar) – campus Lagoa do Sino Buri Brazil; ^3^ Complexo Cultural e Científico de Peirópolis, Universidade Federal do Triângulo Mineiro Uberaba Brazil; ^4^ Bio‐Ambiental Consultoria em Meio Ambiente Ltda Belo Horizonte Brazil; ^5^ Departamento de Medicina Veterinária Universidade Federal de Lavras Lavras Brazil; ^6^ Present address: Department of Earth and Environment Florida International University Miami Florida USA

**Keywords:** curimatá‐pioa, DNA metabarcoding, homing, hormonal profile, migration drivers

## Abstract

Understanding complex migration patterns, including drivers of partial migration and habitat use, is challenging but essential for conservation, as it determines a species' adaptative capacity in the face of environmental change and anthropogenic threats. Therefore, we investigated the traits of partial migration and spawning use in the long‐distance migratory and ecosystem engineering fish *Prochilodus costatus* (pioa). Data from 200 adult individuals tagged with radio transmitters at their feeding and spawning sites, combined with egg and larvae sampling identified through metabarcoding, revealed strong fidelity to both feeding and spawning sites. Providing a comprehensive overview of the pioa life cycle, this study shows that only fish present at specific upstream sites reproduce, indicating that populations isolated downstream, or whose migrations are blocked for any reason, fail to reproduce. Adults may also remain resident at spawning grounds, but these residents are smaller and have lower body condition, suggesting suboptimal feeding conditions at these sites. Use of spawning grounds was not evenly distributed: approximately half of the tagged fish performed annual upstream reproductive migrations, with 58.6% migrating to Bambuí River and 41.49% to the upper Sao Francisco River basin spawning sites, displaying homing behaviour. Hormone levels, body weight, and condition factor were not related to migration decisions, suggesting that the drivers of partial migration remain unclear. Our findings reveal putative population structuring, site fidelity, habitat use, and partial migratory behaviours shared with other phylogenetically distant fish groups, indicating that these results may be generalizable to other Neotropical migratory species.

## 1 INTRODUCTION

Migration is a highly diverse phenomenon in fish life history, occurring in a wide variety of environments (Lennox et al., 2019; Ohta et al., 2025; Sakamoto, 2025) and exhibiting several different patterns across species (Seyedhashemi et al., 2025; Vu et al., 2022) and populations (Jørgensen et al., 2008; Mather et al., 2025). This variability includes the level of fidelity to home sites (de Pontual et al., 2023; Godoy, 1975) and significant differences in the drivers (Roberts et al., 2024) and patterns of partial migration, including whether both migrants and residents reproduce, or only the migrants do. These drivers and patterns of partial migration determine the adaptation face of environmental changes and threats, the reason why management, and consequently conservation, is utterly relying on understanding migratory patterns (Chapman et al., 2014).

Migratory fish species are typically of high ecological and economical importance (Duponchelle et al., 2021), providing numerous ecosystem services (Pelicice et al., 2023). Despite their significance, fish movement ecology has received less attention compared to other animal taxa (Chapman et al., 2014), with an even greater gap for Neotropical fishes, as most published research has focused on diadromous species from the Northern Hemisphere (Cooke et al., 2011). Although the migratory behaviour of Neotropical fishes has been observed for centuries, several traits remain poorly understood (Harvey & Carolsfeld, 2003), especially those related to partial migration. In Neotropical rivers, Agostinho et al. (2003) reported large schools of migratory species remaining stationary near the transition to Itaipu reservoir during the reproductive season, suggesting that some populations may reproduce without migrating to traditional upstream spawning sites. Understanding this possibility has clear conservation implications: if reproducing in downstream areas is possible, these fish could continue reproducing after river damming; however, if not, they may face severe population declines when isolated. For example, the Mandi (*Pimelodus maculatus* Lacepède 1803), formerly considered a migratory species, has adapted and thrives in dammed rivers (Santos et al., 2013), showing itself to be a non‐obligate migrant. Conversely, migration behaviour is considered essential for many other species, as significant population declines have been observed in fragmented rivers (Agostinho et al., 2007).

Although this partial migration behaviour is common among freshwater fish, most studies focus on species where both migratory and non‐migratory individuals reproduce (Shaw & Levin, 2011). Less is known about those species with well‐defined breeding sites, where only individuals living at these sites or those migrating to them reproduce, while the remainder forgo reproduction. This form of partial migration with intermittent reproduction suggests a decision‐making process influenced by individual differences (Chapman et al., 2011). For species in the Northern Hemisphere, pre‐migration conditions such as body condition (Birnie‐Gauvin et al., 2021) and size (Lavender et al., 2024; Thériault & Dodson, 2003) can influence the decision to migrate (Jørgensen et al., 2008) and the migration distance (Keefer et al., 2009). Various endocrine factors also influence migration. For example, higher cortisol levels increase the likelihood of initiating downstream migration in *Salmo* species (Birnie‐Gauvin et al., 2019), and 17β‐estradiol levels change significantly during the spawning migration (Slater et al., 1994). However, most of this knowledge is restricted to temperate diadromous species, and the role of hormonal profiles on migration has rarely been investigated in potamodromous Neotropical fishes (see Arantes et al., 2010).

Among the most common migratory fishes in Neotropical river basins are the species of the *Prochilodus* genus. Many of them are highly important for South American fisheries, sometimes accounting for more than a half of the total biomass or fishery yields in some rivers (Bowen, 1983). *Prochilodus* play key roles in nutrient cycling and are considered ecosystem engineers (Flecker, 1996), converting poor nutrient sources (sediment and periphyton) into protein. However, yields have declined in recent decades (Agostinho et al., 2007; Duque et al., 1998). Due to their ecological and economic importance, studies on Neotropical fish migration have focused on *Prochilodus* species in rivers such as the São Francisco (Godinho & Kynard, 2006; Lopes et al., 2018; Lopes, Pompeu, et al., 2019; Peressin et al., 2024), Jequitinhonha (Silva et al., 2021), Paraná (Avigliano et al., 2017; Espinach‐Ros et al., 1998), Sinos (Pesoa & Schulz, 2010), Orinoco (Duque et al., 1998), and Amazon (Silva & Stewart, 2017). *Prochilodus costatus* Valenciennes, 1850, is endemic to the São Francisco River basin and known locally as ‘curimatá‐pioa’ or simply ‘pioa’, has been studied over the past decade in the upper São Francisco River basin. These studies have provided valuable information on its life cycle and natural migratory patterns (Lopes et al., 2018, Lopes, Pompeu, et al., 2019; Lopes, Alves, et al. 2019; Pompeu et al., 2023), making them a good model for deepening knowledge about Neotropical fish migration patterns. However, at least three new power plants are planned in this stretch of the São Francisco River, posing serious threats to this remnant population (Lopes et al., 2025).

Similar to the *Prochilodus* species, migratory fish populations are declining worldwide due to habitat loss, physical barriers, overexploitation, and climate change (Duponchelle et al., 2021; Hauser et al., 2024). The lack of understanding of migratory behaviour has led to ineffective conservation and mitigation measures (Pelicice et al., 2015). To maintain migratory fish populations and predict their responses to anthropogenic disturbances, it is essential to understand their life cycle, including factors shaping partial migration (Roberts et al., 2024). However, despite considerable efforts to study Neotropical fish migration, detailed knowledge about their life cycle, including site fidelity, migration trade‐offs, and drivers of partial migration, remains scarce. For this reason, this study uses pioa as a model to (i) quantify the most relevant spawning sites and assess whether spawning site fidelity occurs; (ii) assess whether hormonal profile, body weight, and body condition factor influence migration decisions and distances; (iii) determine if there are resident fish at the spawning site, and if so, why some fish undertake extensive migrations. We also provided a synthesis of the specie's life cycle.

## 2 METHODS

### 2.1 Study area

**FIGURE 1:**
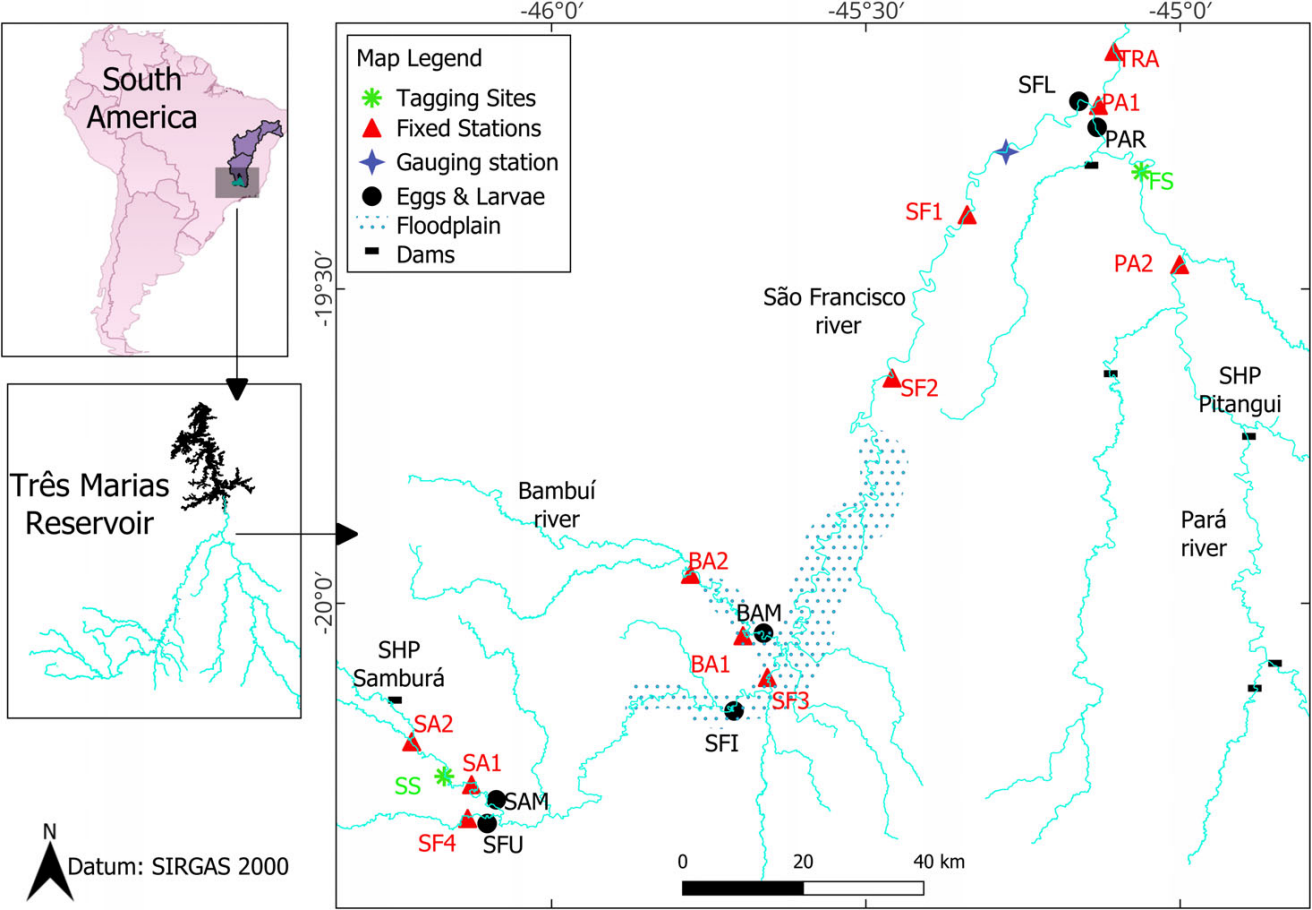
Locations of the pioa (*Prochilodus costatus*) tagging sites (FS = feeding site; SS = spawning site), gauging station for environmental variables, fixed telemetry stations (legends as in Figure 2), eggs and larvae sampling sites (according to Table 2), and dams (SHP, small hydropower plants) in the upper São Francisco River basin, upstream of the Três Marias reservoir, Brazil, from September 2019 to April 2022. The purple area on the South America map represents the entire São Francisco River basin.

To evaluate reproductive migration, residence at spawning sites, spatial distribution of adults in the basin and site fidelity, two tagging sites were selected in a free‐flowing remnant, one representing a feeding site and the other representing spawning sites, both according to Lopes et al., (2018) and Lopes, Pompeu, et al., (2019). These sites are situated in the upper São Francisco River basin, Brazil, upstream of the Três Marias hydroelectric dam reservoir (Figure 1). The main stem of the river stretches 450 km from its headwaters to the 1090 km^2^ reservoir formed by the dam. The upper São Francisco basin covers an area of 26,680 km^2^ and is fed by significant tributaries such as the Bambuí, Samburá and Pará rivers, among others. The Pará River basin has several hydroelectric dams in tributaries and main stem, whereas Samburá River also has a small hydropower plant (SHP). Together, SHP Samburá, located on the Samburá River, and SHP Pitangui, located on the Pará River – both situated upstream of the tagging sites – represent the upper migration limit in these rivers (Figure 1). Floodplain lagoons, recognized as crucial growth areas for migratory species, are abundant in the region (Moreira, Peressin, Lopes, & Pompeu 2023). The climate is classified as tropical wet (Cwa), with a rainy season from October to March and an annual average precipitation of 1372 mm (Lopes et al., 2018; Lopes, Pompeu, et al., 2019).

### 2.2 Adult fishes

#### 2.2.1 Tagging and hormonal profile

Based on previous knowledge of pioa migration in the basin, adult pioa leave the feeding sites from late September to early October, reaching spawning sites until December (Lopes et al. 2018; Lopes, Pompeu, et al., 2019). Therefore, pioa were captured at a feeding site in September 2019 and August/September 2020, and at a spawning site in September/December 2019, August/September 2020 and December 2020. These tagging data aimed to tag individuals at both sites prior to the migration onset, as well as during peak spawning site aggregation in December. Pioas at the feeding site were tagged on the Pará River (19° S 16′49.50″, 45° W 4′42.40″), 16 km upstream from its confluence with the São Francisco River (Lopes, Pompeu, et al. 2019), where their population exhibits the highest genetic diversity in the study area (Ludwig et al., 2023). Those at the spawning site were tagged on the Samburá River (20° S 17′43.60″, 46° W 7′4.30″), also 16 km upstream from its confluence with the São Francisco River (Figure 1).

One hundred adult fish were tagged at each site, totalling 200 fish. Captures were conducted at both the feeding and spawning sites by local professional fishermen using cast‐nets to reduce stress and physical injuries to the fish. After capture, all fish were placed in floating mesh cages installed in the river. Subsequently, the fish were individually transported using dip‐nets to the surgical station. Procedures for anaesthesia, surgery, tagging with radio transmitters (Lotek MCFT2–3EM–M, 10 g in air, with an estimated battery lifetime of 659 days and a burst rate of 6 s) and fish recovery followed the routine described in Peressin et al. (2021). All procedures were conducted with capture permits (SISBIO 10327) and approval from the ethics committee of the Federal University of Lavras (CEUA UFLA 003/2019).

To assess the drivers of partial migration, after anaesthesia and before tagging, fish were measured for weight and length, and approximately 1 mL of blood was drawn by caudal vessel puncture for physiological analysis, using a syringe and disposable needles with EDTA anticoagulant. The collected blood was stored in Eppendorf tubes placed in a thermal box with ice. After tagging and recovery, all the fish were released at the same site where they were captured.

Daily, at the end of sampling, blood samples were centrifuged at 1000 rpm for 5 min, and plasma was separated by pipetting and frozen. Levels of 17β‐estradiol (pg/mL), testosterone (pg/mL), and cortisol (μg/dL) in plasma were measured using enzyme‐linked immunosorbent assays (ELISA kit – USA Diagnostica), with absorbance read by spectrophotometry at 450 nm.

#### 2.2.2 Monitoring

Eleven fixed telemetry stations were positioned across the study area for comprehensive coverage. These stations spanned locations, including the transition from lotic to lentic environments in the Três Marias reservoir (TRA), the main channel of the São Francisco River (SF1, SF2, SF3 and SF4) and the tributaries Pará (PA1 and PA2), Bambuí (BA1 and BA2) and Samburá (SA1 and SA2) rivers. Together, they covered approximately 530 river kilometres. The first station in each tributary was situated near its confluence with the São Francisco River (Figure 1). Each station comprised two antennas with five elements, one directed upstream and the other downstream, positioned at a height of 5–7 m. They were connected to a Lotek SRX‐DL receiver and powered by batteries equipped with solar panels.

Boat tracking was conducted in November 2019, August 2020, and February 2021 along the Pará and São Francisco rivers, between the fixed stations PA2 and TRA, passing through PA1. This covered a segment spanning approximately 62 km, encompassing the area where fish were tagged at the feeding site. The objective of these boat‐tracking activities was to identify fish that remained undetected at fixed stations but were still active. This was facilitated by motion‐sensing capabilities in the transmitters, which alerted when fish remained stationary for over 24 h. However, conducting similar tracking at the spawning tagging site was unfeasible due to impassable obstacles for the boat. During the boat tracking, fish were detected using a three‐element antenna connected to a Lotek SRX‐600 receiver.

Detectability tests were performed after the installation of all fixed stations and prior to each boat tracking, using test transmitters representing all five radio frequencies employed in the study. For these tests, active transmitters were placed on the riverbank and in the main river channel, approximately 50 m from the antennas, and left for 5 min, enough time for the receiver to scan all five frequencies at least five times. The receivers were monitored in real time to confirm detection. Detectability was considered satisfactory if all transmitters were detected with a signal strength above 150.

### 2.3 Eggs and larvae sampling

Aiming to assess the spatial distribution of spawning to determine whether reproduction is restricted to fish present at spawning sites or shared with fish that remain resident at feeding site, eggs and larvae were sampled at six sites distributed throughout the study area. Three sites were located along the main channel of the São Francisco River (upper = SFU, intermediate = SFI, and lower = SFL), and three additional sites were located near the confluences of Sambura (SAM), Bambuí (BAM), and Pará (PAR) rivers with the São Francisco River (Figure 1).

Sampling was conducted by previously trained local fishermen every 3 days between November 2019 and February 2020, totalling 244 samples. Of these, 41 were collected at each sampling station (SFI, SFL, BAM, and PAR), whereas 40 were collected at SFU and SAM due to a flooding event that prevented sampling on 29 February 2020. This period was chosen as it encompasses the previously known reproductive period of migratory fish in this basin (Bazzoli, 2003; Lopes, Pompeu, et al. 2019). Collections took place in the main flow between 4:00 and 5:30 PM, using a conical ichthyoplankton net with a mesh‐size of 500 μm and an adapted flowmeter for calculating the filtered volume. The net was submerged to a depth of approximately 20 cm in the flowing water for 10 min, with water temperature measured during each sample.

These samples of ichthyoplankton fixed in absolute alcohol were identified at the species level using DNA metabarcoding, following the high‐throughput Illumina methods described in Pompeu et al. (2023). All eggs were identified through metabarcoding, whereas larvae were first grouped into morphospecies using a stereomicroscope. Subsequently, representative specimens from each group were selected for species confirmation through barcoding. A spawning event was defined as an abundance peak of eggs, measured from the relative read abundance (RRA), occurring at least in one sampling station, with a minimum interval of 6 days among events.

### 2.4 Data analysis

Telemetry records underwent validation based on sequence and signal strength. Sequences with fewer than three signals and/or with only low‐intensity signals (below 100 on a scale ranging from 0 to 255) were discarded due to a high probability of external interference with the receiver. A sequence of regular signals with increasing strength (usually above 150) detected by the same fixed station was considered a valid record. Records detected at time intervals shorter than the interval between two signal emissions by the tags (burst rate) were also discarded. A similar procedure was employed to validate records obtained through boat tracking. The number of active fish tagged at the feeding site was calculated based on individuals registered at least once at a fixed station or recorded as active (according to motion sensing) during at least one boat‐tracking survey. Fish detected as inactive based on motion sensing during all boat‐tracking events and with no detections at fixed station were excluded from the analysis. The number of active fish was calculated only for the feeding site because boat tracking was not feasible at the spawning site.

The onset of migration was defined as the first recorded detection of a fish after leaving the tagging site, corresponding to its passage at the first fixed station downstream or upstream of the tagging site. Migration destination was defined as the farthest upstream site reached, that is, the most distant fixed station where a fish was detected. The return migration from spawning to feeding site was identified by the first detection at a fixed station during downstream movements after reaching the farthest upstream point. If a fish initiated a new upstream movement after returning downstream, it was considered a new spawning migration. This process was repeated until the transmitter was no longer active. The migratory period for individual fish was defined as the number of days between the departure date from the tagging site and arrival at the farthest upstream site reached. For fish tagged at the feeding site, we tested whether the migration distance and the migratory period were correlated, using Spearman's rank correlation.

Based on the classification of Neotropical migratory fish proposed by Agostinho et al. (2003), we considered those fish tagged at the feeding site that migrated more than 100 km upstream as long‐distance migrants. Thus, only individuals reaching the SF2 station or farther were classified as long‐distance migrants. Fish that remained at this tagging site but were active during boat tracking, as well as those that moved less than 100 km upstream were considered non‐migratory.

Considering only fish tagged at the feeding site, we used generalized linear models (GLMs) to assess drivers of partial migration. In the first model we coded the fate of each individual as non‐migratory (0) or long‐distance migrants (1) to fit a binomial GLM with logit‐link function. Independent variables were body weight, Fulton's body condition factor, plasma 17β‐estradiol, testosterone, and cortisol. Fulton's condition factor was calculated for all tagged fish using the formula BW/SL^3, where BW represents body weight and SL represents standard length.

We have also investigated if the predictors mentioned above influenced the distance migrated by long‐distance migrant fishes. So, we fitted a second model, using gamma GLM with log‐link (GLM 2), to test whether upstream migration distance was influenced by the same variables. Additionally, we used gamma GLMs to test for differences in body weight (GLM 3) and Fulton's condition factor (GLM 4) between fish tagged at spawning versus feeding sites, restricting the analysis to fish tagged in August/September. For the first and second models, we applied a stepwise procedure in which predictors were both added or excluded to retain the model with the lowest AIC. To assess multicollinearity, we calculated variance inflation factors (VIFs). Following Jones et al. (2022), values above 5 are considered problematic, whereas values close to 1 indicate no evidence of collinearity.

For fish tagged at the spawning site, we applied χ^2^ test to evaluate whether the proportion of individuals leaving the area differed between those tagged in August/September and in December. Finally, fish recaptured by fisherman were included in the analyses only if the capture occurred after the end of the migratory window. All statistical analysis was performed using R software (R Core Team, 2025).

## 3 RESULTS

### 3.1 Adult fishes

#### 3.1.1 Migratory movements

##### Fish tagged at feeding site

**FIGURE 2:**
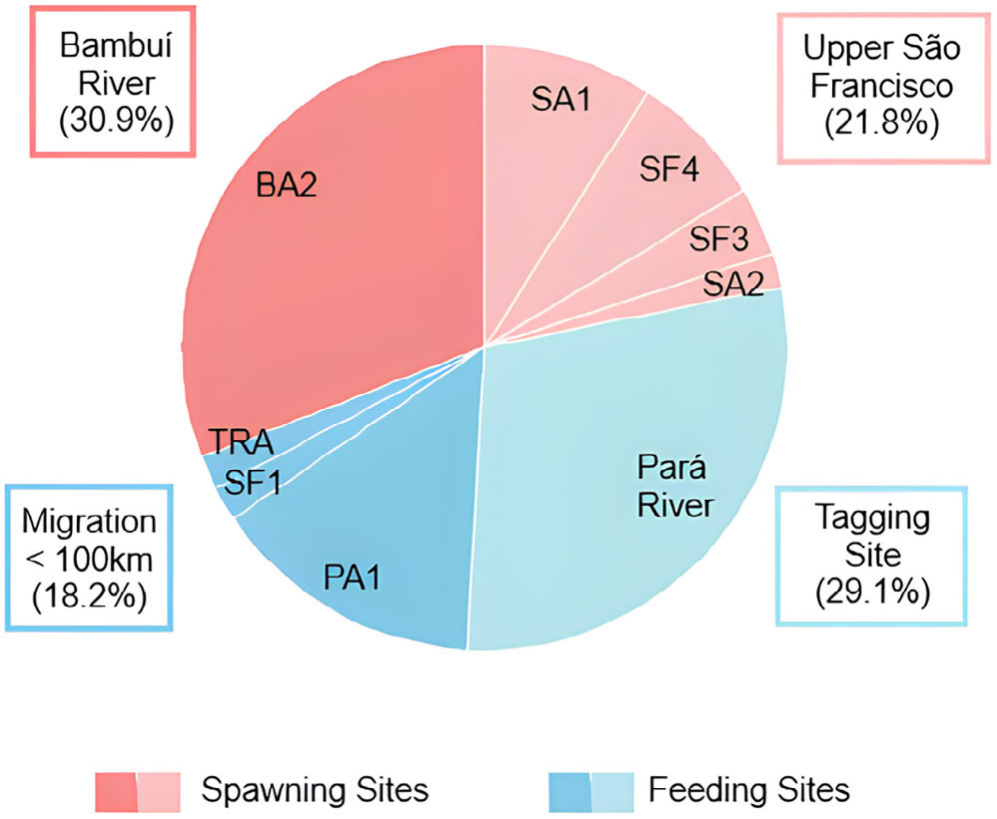
Destination (the most distant fixed station reached and measured from the tagging site) of fish tagged at a feeding site (Pará River) in 2019 and 2020. Fish that reached the spawning sites performed long‐distance upstream reproductive migrations (>100 km). Fixed telemetry stations: TRA – transition from the lotic stretch to the Três Marias reservoir; PA1 – confluence of the Pará River with the São Francisco River; BA2 – Bambuí River, 50 km upstream from BA1; SA1 – Samburá River, 8 km upstream from its confluence with the São Francisco River; SA2 – Samburá River, 34 km upstream from SA1; SF1 – main channel of the São Francisco River, 50 km upstream from the confluence with Pará River; SF3 – main channel of the São Francisco River at the confluence with the Bambuí River, 162 km upstream from SF1; SF4 – São Francisco River upstream from the confluence with the Samburá River. Only fish that were detected at least once at a fixed station, or detected as active based on motion sensing during boat tracking, were included.

Among the 100 fish tagged at the feeding site over both years, 19 were never detected after release, 23 were recorded only as inactive, and 4 were reported as captured by local fisherman. Of the remaining 55 detected and active fish, 52.7% (*N* = 29) undertook long‐distance upstream migrations toward the spawning sites. They moved 16 km downstream in the Pará River, then headed towards the main channel of the São Francisco River, initiating upstream migrations from that point. Distances travelled ranged from 100 to nearly 400 km upstream, with destinations including the upper São Francisco and its headwater branch, the Sambura River (*N* = 12, 41.4%), or the Bambuí River, another major tributary (*N* = 17, 58.6%). Most of the fish that entered Bambuí River reached BA2 fixed station, located 50 km upstream from its confluence with the São Francisco River (Figure 2; Table 1). These long‐distance migratory fish tagged in 2019 left the feeding site toward the spawning grounds between 22 september and 21 november 2019, while those tagged in 2020 migrated between 7 and 26 october 2020. The other 47.3% (*N* = 26) of detected and active fish did not undertake long upstream migrations. They either remained near the tagging site or performed short migrations, covering distances of at least 16 (*N* = 8), 28 (*N* = 1) and 66 km (*N* = 1). There was no significant correlation between migration distance and the duration of the migratory period (rs = 0.1; *p* = 0.69).

**TABLE 1:** Tagging site, tagging period, number of tagged fish, standard length (range and ± SD, mm), body weight (range and ± SD, g), tag ratio (tag weight divided by fish weight), number of fish detected as active after tagging (*N*_a_, *not calculated for fish tagged at spawning site), migration destination, number of fish by migratory destination (*N*_d_), total number of fish performing migration, percentage of fish that left the tagging site, and period during which the fish left the tagging sites (displacement window).

Tagging site	Tagging period	*N*	Standard length	Body weight	Tag ratio (%)	*N*_a_*	Destination of migration	*N*_d_	Total	%	Displacement window
Feeding	Sep 03^rd^–20^th^, 2019	50	325.38 (276–404 ± 32.43 mm)	829.4 (530–1,400 ± 245.96 cm)	0.71–1.89	30	Bambuí (280 km)	9	19	63.3	Sep 22^th^, 2019–Jan 25^th^, 2020
							São Francisco (16–365 km)	9			
							Samburá (362 km)	1			
Feeding	Aug 03^rd^–Sep 03^rd^, 2020	50	334.68 (272–430 ± 34.74 cm)	846.2 (505–1,475 ± 221.66 cm)	0.68–1.98	26	Samburá (362–392 km)	5	21	80.8	Ago 12^th^, 2019–Oct 26^th^, 2020
							Bambuí (280 km)	8			
							São Francisco (16–228 km)	7			
							Reservoir (28 km)	1			
Spawning	Sep 23^th^ −29^th^, 2019	25	324.64 (262–432 ± 39.96 cm)	803 (505–1,750 ± 296.3 cm)	0.57–1.98	‐	São Francisco (8–142 km)	10	12	48	Nov 29^th^, 2019–Mar 02^nd^, 2020
							Bambuí (192 km)	1			
							Pará (354 km)	1			
Spawning	Dec 04^th^–08^th^, 2019	26	314.54 (269–393 ± 34.16 cm)	698.27 (500–1,265 ± 202.64 cm)	0.79–2	‐	São Francisco (8–142 km)	13	19	73.1	Dec 11^th^, 2019–Mar 28^th^, 2020
							Bambuí (192km)	2			
							Pará (354 km)	2			
							Reservoir (366 km)	2			
Spawning	Aug 28^th^–Sep 11^th^, 2020	25	333.2 (290–400 ± 28.62 cm)	754.2 (500–1430 ± 222.03 cm)	0.7–2	‐	São Francisco (8 km)	4	5	20	Sep 13^th^, 2020–Feb 27^th^, 2021
							Pará (354 km)	1			
Spawning	Dec 01^st^–04^th^, 2020	24	323.71 (289–394 ± 26.09 cm)	717.5 (500–1,150 ± 177.98 cm)	0.87–2	‐	São Francisco (8–304 km)	9	13	54.2	Dec 02^th^, 2020–Apr 15^th^, 2021
							Bambuí (142km)	1			
							Pará (354 km)	2			
							Reservoir (366km)	1			

##### Fish tagged at spawning site

**FIGURE 3:**
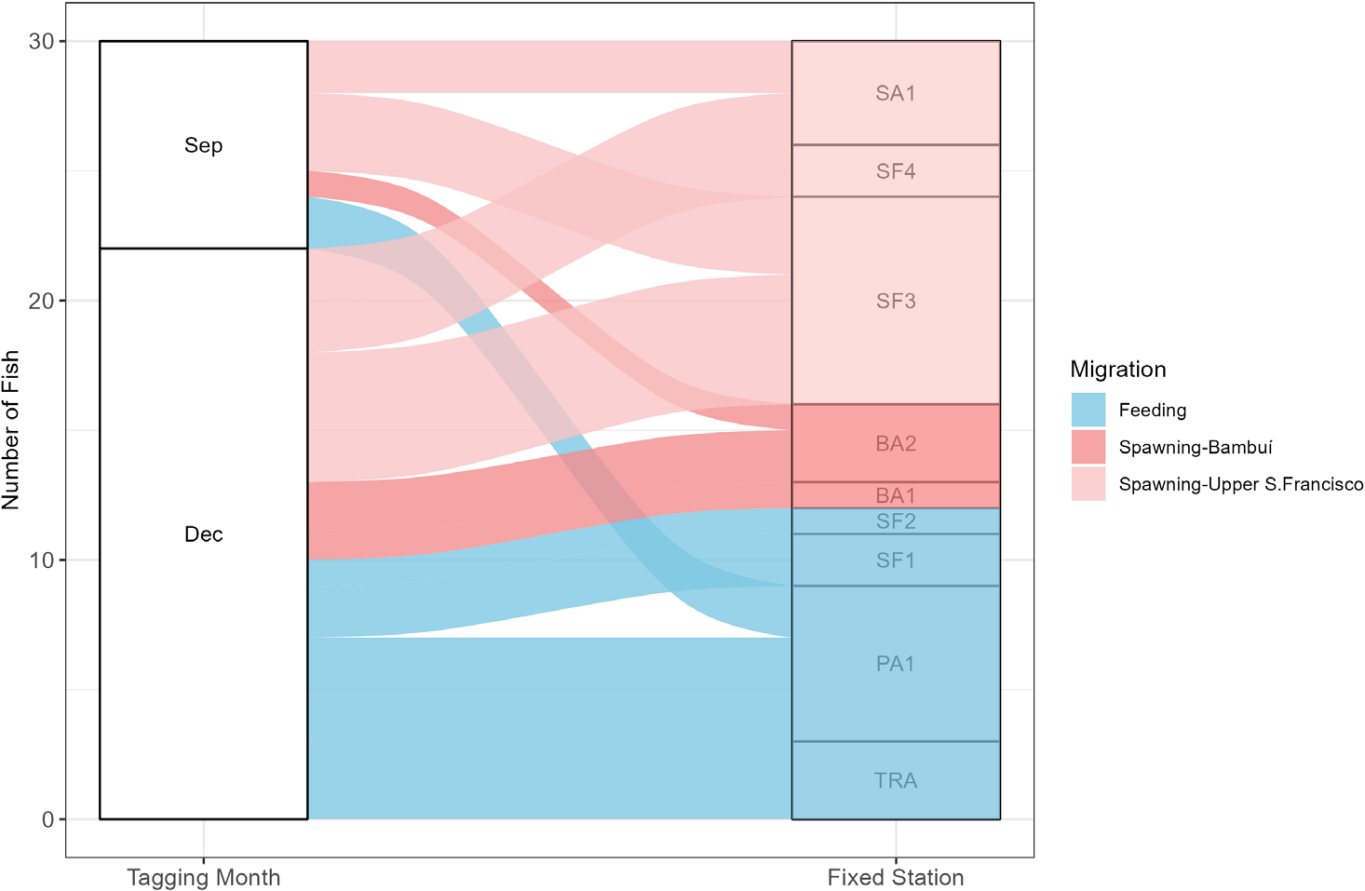
Destination (most distant fixed station reached and measured from the tagging site) of fish tagged at a spawning site (Samburá) in September (Sep) and December (Dec) of 2019 and 2020. The thickness of the lines indicates the number of fish that moved in each direction. Fixed telemetry stations according to Figure 2. BA1 – confluence of the Bambuí River with the São Francisco River; SF2 – main channel of the São Francisco River, 50 km upstream of SF1. Only fish that undertook movements and were detected at least once at a fixed station were included.

Because boat tracking was not possible near the spawning site, we excluded from the migration destiny quantification and from the χ^2^ test any fish reported as captured by local fishermen at the tagging site and undetected at any fixed station (*N* = 4). As a result, 96 tagged fish over both years in the spawning site were included in the analysis. Considering fish tagged in 2019 and 2020, among the 47 individuals tagged in September, 36.2% (*N* = 17) left the tagging site, whereas among the 49 tagged in December, 65.3% (*N* = 32) did the same. The proportion of fish that left the spawning site was significantly higher among those tagged in December (χ^2^: test statistic = 8.15; *p* < 0.01). These fish had various downstream destinations, ranging from 8 to almost 400 km downstream of the tagging site and 22 km upstream (Figure 3; Table 1).

#### 3.1.2 Site fidelity

**FIGURE 4:**
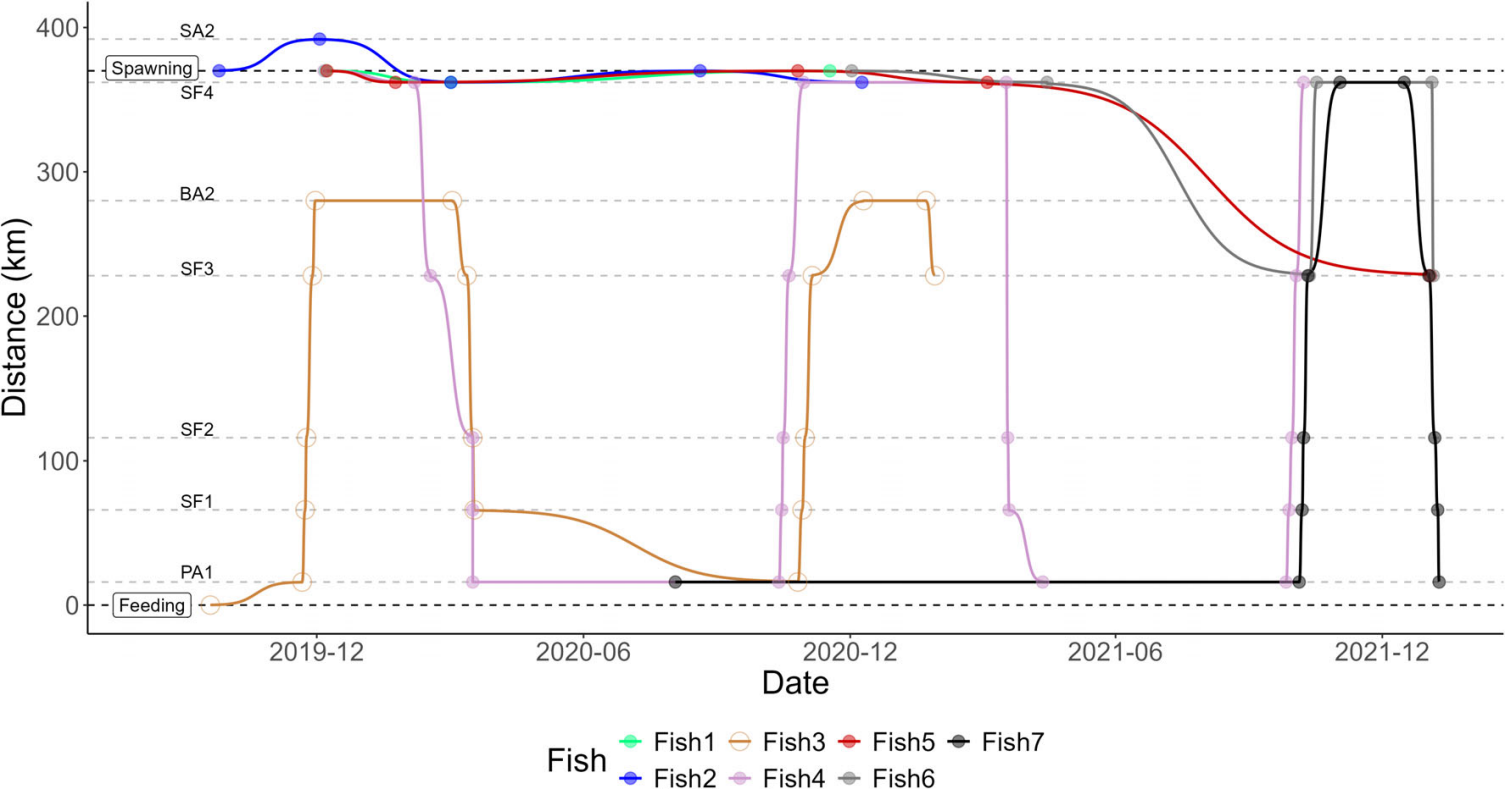
Movements of fish monitored for at least two consecutive spawning seasons (feeding – location of feeding tagging site; spawning – location of spawning tagging site; fixed station abbreviations as in Figure 2). Both fish tagged at feeding site (3 and 7) and one from spawning site (Fish 4) performed long‐distance migratory movements (>100 km).

Eight fish had their movements monitored for at least two consecutive spawning seasons: two tagged at the feeding site and six at the spawning site. One of them, tagged in the spawning site (not depicted in Figure 4), did not undertake migration in either of the 2 years. In another case (Fish 7), the fish tagged at the feeding site in 2020 did not migrate in the first year after tagging but did so in the subsequent spawning season.

In the remaining six cases, there was a visit to the same spawning site in consecutive spawning seasons. In one instance (Fish 4), the fish tagged at the spawning site in December 2019 moved approximately 350 km downstream, reaching the Pará River, where it remained until the next spawning season when it revisited the Samburá River spawning site in November 2020. The fish then repeated this movement, travelling downstream to the Pará River and subsequently migrating back to the Samburá River, demonstrating interannual fidelity to both the spawning and feeding sites.

#### 3.1.3 Internal state and physiological drivers of migration

According to the first GLM, fish that undertook long‐distance upstream reproductive migrations did not differ significantly from those that remained at the feeding site in terms of body weight, body condition factor, plasma 17β‐estradiol, testosterone, or cortisol. However, 17β‐estradiol and body weight were retained in the final selected model (Table 2). The VIF value (1.41) indicated no collinearity among independent variables.

**TABLE 2:** Parameters for generalized linear models (GLMs 1–4) showing only the final models selected after stepwise regression (both directions) based on the lowest Akaike information criterion (AIC) values.

Parameter (GLM binomial)	Estimate	Standard error	*z* statistic	Probability (*p*)	Degrees of freedom	AIC
GLM 1					51	73.99
Intercept	−122.84	1.18	−1.04	0.29		
Body weight	0,00	0.00	1.51	0,13		
17β‐estradiol	−0,00	0.00	−1.79	0,07		

Parameter (GLM Gamma)	Estimate	Standard error	*t* statistic	Probability (*p*)	Degrees of freedom	AIC
GLM 2					26	336.7
Intercept	0.29	1.63	0.18	0.86		
17β‐estradiol	0.00	0.00	2.18	0.04*		
Body condition factor	1.20	0.70	1.71.	0.10		
GLM 3					198	2716.6
Intercept	0.00	0.00	34.03	<0.01*		
Body weight	0.00	0.00	2.88	<0.01*		
GLM 4					198	47.32
Intercept	0.44	0.00	81.52	<0.01*		
Body condition factor	0.03	0.01	3.59	<0.01*		

*Note*: *significant values (*p* < 0.05).

**FIGURE 5:**
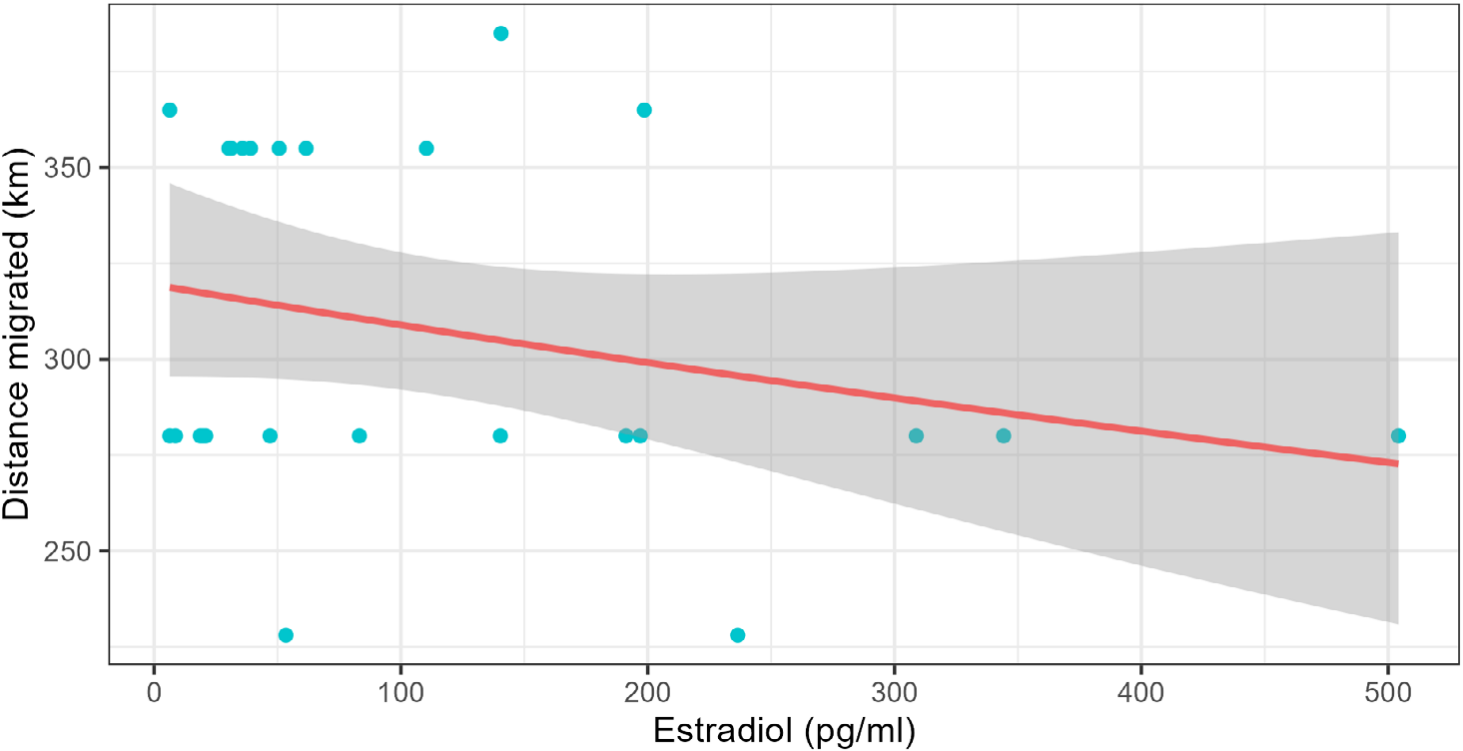
Relationship between migrated distance and 17β‐estradiol levels in fish tagged at the feeding site in August/September 2019 and 2020 that performed long‐distance upstream reproductive migrations (>100 km).

The second GLM indicated that migration distance in upstream reproductive migration was negatively associated with 17β‐estradiol (Figure 5), but not with body weight, body condition factor, plasma cortisol, or testosterone (Table 2). The VIF value (1.02) indicated no collinearity among independent variables.

**FIGURE 6:**
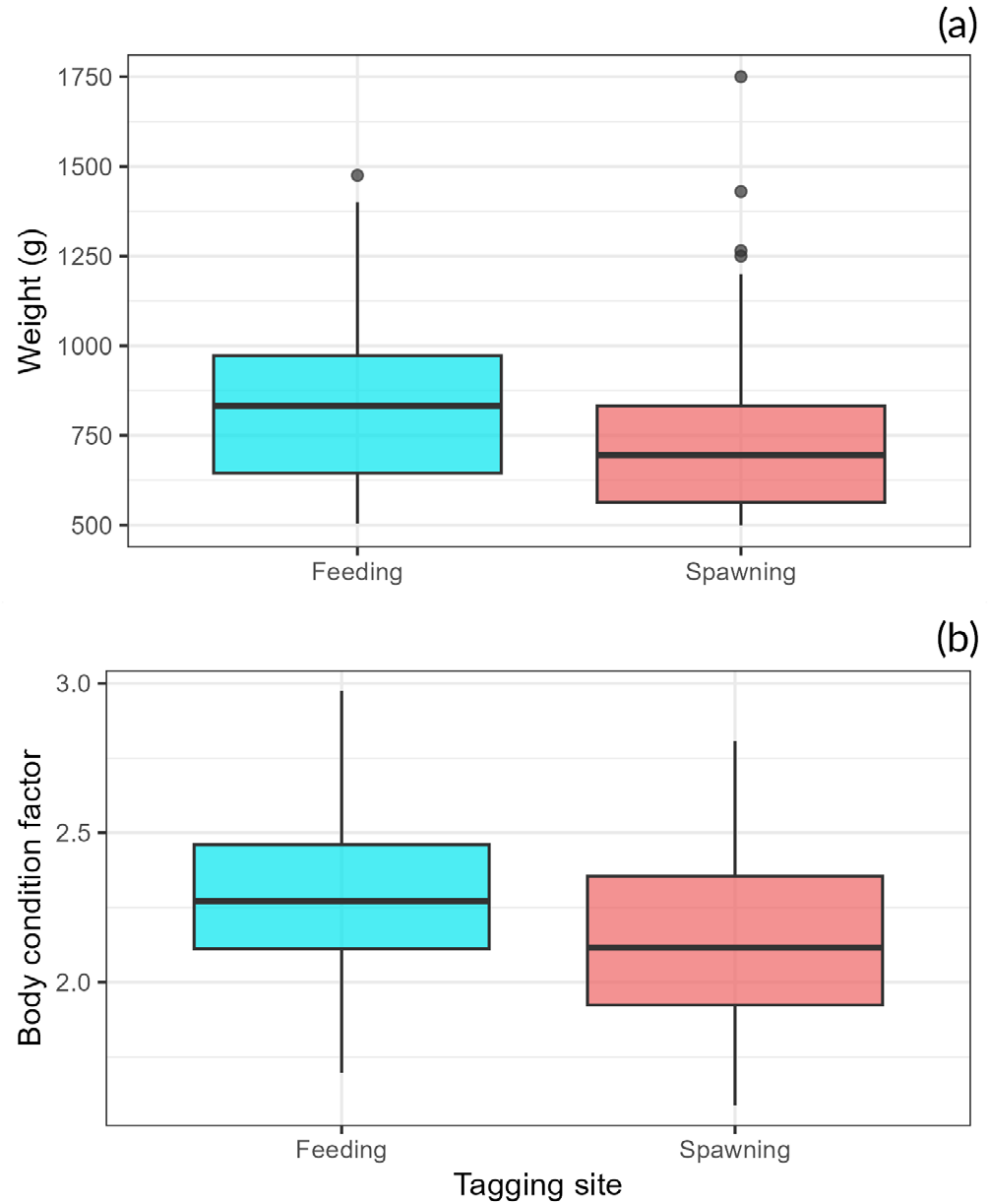
Boxplots of body weight (a) and Fulton's body condition factor (b) for fish tagged at feeding and spawning sites in August and December 2019 and 2020.

Finally, the third and fourth models evidenced, respectively, that body weight (Figure 6a) and body condition factor (Figure 6b) of fish tagged in August/September were higher at the feeding site compared to the spawning site (Table 2).

### 3.2 Eggs and larvae: Spatial distribution of spawning

Considering all 118 pioa eggs identified through metabarcoding, 67% were sampled in upper sites (SAM and SFU), 33% in intermediate sites (SFI and BAM), and none in the lower sites (PAR and SFL). Additionally, 49 larvae were sampled in the lower sites of the Bambuí and São Francisco rivers. Based on eggs and larvae samples, at least five spawning events of pioa occurred during the study period, between 10 November 2019 and 29 February 2020. These events were observed only in the upper part of the basin (sites SAM, SFU, and SFI) and in the Bambuí tributary (BAM), which had the highest egg densities. Larvae were detected in the Bambuí River and in the lower course of the basin (SFL) (Table 3).

**TABLE 3:** Eggs and larvae of pioa (*Prochilodus costatus*) identified by metabarcoding.

	Eggs and larvae sampling sites					
	SAM	SFU	BAM	SFI	PAR	SFL
Mean eggs density (N/m^3^)	0.32	0.49	2.21	0.32	0	0
Number of breeding events	4	3	4	5	‐	‐
Breeding period (range)	1 Dec 26 Feb	24 Jan 26 Feb	10 Nov 17 Feb	4 Dec 29 Feb	‐	‐
Number of sampled larvae			10			39

*Note*: Sampling locations include the upper (SFU), intermediate (SFI), and lower (SFL) sections of main channel of the São Francisco River, as well as the tributaries Pará River (PAR), Bambuí River (BAM), and Samburá River (SAM) near their confluences with the Sao Francisco.

## 4 DISCUSSION

Our results revealed a remarkable case of partial migration, in which only a fraction of population migrates and reproduction occurs exclusively among individuals reaching specific reproductive grounds, as evidenced by quantitative assessment of eggs and larval distribution. Although some individuals remain resident at spawning sites – because most fish tagged in September at the spawning site never left this place – these residents were smaller and exhibited lower body condition factors. Long‐distance migrants tagged at feeding site moved upstream to the Bambuí River and the upper São Francisco basin, including the Samburá tributary, but these sites were not equally used. Homing behaviour to the spawning sites was confirmed by individuals recorded in two consecutive reproductive migrations that returned to the same sites, suggesting the existence of putative segregated populations. Combined with the species' known fidelity to feeding sites (Lopes et al., 2018), these findings demonstrate both reproductive and feeding homing. Drivers of partial migration were not identified, as hormonal profile, body weight, and body condition factor at tagging were unrelated to the migration decisions. However, 17β‐estradiol levels were inversely correlated with migrated distance.

### 4.1 Migration patterns and site fidelity

These types of migration patterns, in which putatively segregated populations with fidelity to different spawning sites share the same feeding sites, are also observed in phylogenetically distant species inhabiting vastly different aquatic environments, such as diadromous semelparous and iteroparous fish in the Northern Hemisphere (Brönmark et al., 2014), large Amazonian catfish (Duponchelle et al., 2016), and oceanic species like bluefin tuna (Block et al., 2005). Fidelity behaviour to spawning sites has also been reported for adult *Prochilodus argenteus* Spix & Agassiz (1829) in the São Francisco River (Godinho & Kynard, 2006), and for *Prochilodus lineatus* (Valenciennes, 1837) in the Paraná River basin (Godoy, 1975), suggesting that this behaviour may be widespread within the *Prochilodus* genus.

However, it is unknown whether the location they return to is the same as where they were born, as seen in salmonids (Klemetsen et al., 2003). This inference is challenging because *Prochilodus* species, like many other Neotropical migratory species, release their eggs into the water, and these eggs drift with the river's flow (Sato et al., 2003). This is quite different from salmonids, which protect their eggs in nests on the riverbed (Fleming, 1996). Consequently, the eggs of *Prochilodus* hatch far from the spawning sites, preventing the imprinting of their migratory route, as seen in salmonids (Putman et al., 2013). Future research should aim to understand the mechanisms of selection of feeding sites by juveniles and of spawning sites by adults during their first spawning.

Because the proportion of fish leaving the spawning site (Samburá River tagging site) after tagging was significantly higher among those tagged in December than September, and given that the reproductive upstream migration in the region extends from late September or October to December (Lopes et al., 2018; this study), we assume that most fish tagged in September are residents at the spawning site, as the majority did not leave this site. Simultaneously, we suggest that many fish tagged in December are individuals that feed elsewhere in the basin and congregate at the spawning during the reproductive season.

These fish that shared the same spawning site in December likely originate from multiple feeding sites across the basin, as postspawning movements indicated returns to multiple locations, including the Três Marias reservoir – the most distant destination, nearly 400 km downstream – as well as to nearby areas. The minimum recorded distance of 8 km corresponds to individuals detected only at station SA1, the first downstream station. However, these fish may have had diverse destinations within the basin, because the next downstream station (SF3) is 142 km away. For example, one individual was captured by local fishermen in the Patos River, a tributary of the São Francisco River, whose confluence is located 58 km downstream from the spawning site. Furthermore, based on the author's personal observations and reports from local fishermen, adults of this species are widely distributed throughout the basin, except in low‐order streams. For these reasons, we propose that the entire basin functions as a feeding area for the species.

Although only four fish tagged at the feeding site (4%) were reported as captured by local fisherman, 20 of these fish (20%) were never recorded again after tagging. Considering that boat tracking at the feeding site covered the entire stretch between two fixed stations, the most likely explanations for this are fishes being caught by fishermen but no reported or tag failure. However, because all tags were tested before implantation, and the site is a traditional spot of recreational and professional fisheries, we suggest that the actual capture rate may be significantly higher than reported. Fishermen commonly avoid reporting catches due to fear of punishment. Additionally, 23% of the fish tagged at the feeding site were only detected as inactive in boat tracks, based on motion sensing, which could indicate mortality due to surgery, tag expulsion, or fish being caught and their tags discarded along riverbanks or thrown into the water. For instance, one of the captures was recorded because a tag was found on a riverbank, most likely as a result of fishing activities. Some natural mortality, or deaths mediated by natural predators who would leave the carcasses/tags on the river banks is also possible, but we have no evidence that this occured.

### 4.2 Drivers of partial migration

The species exhibited a clear pattern of partial migration, as fish that feed outside of spawning sites must annually decide whether to migrate. However, factors such as body weight, body condition factor, plasma 17β‐estradiol, testosterone, and cortisol did not influence the decision. Although Birnie‐Gauvin et al. (2019) demonstrated that elevated cortisol levels increase the likelihood of downstream migration in juvenile salmonids, cortisol has not been found to be a decisive factor in upstream migration (Kostin et al., 2024).

In contrast, migration distance was inversely correlated with 17β‐estradiol levels. Because this hormone is typically higher in females than in males (Abdollahpour et al., 2024), it is possible that fish with lower 17β‐estradiol levels, which migrate longer distances, are males, whereas females probably migrate shorter distances to nearer sites. Although sex‐related differences in migration patterns have also been reported for salmonids (Lavender et al., 2024), we were not able to determine sex and gonadal stage because most fish presented gonads at early stages of maturation at tagging, making it unfeasible to distinguish between within‐sex and across‐sex effects. Therefore, the potential cause–effect relationship between migration distance and 17β‐estradiol within each sex in Neotropical fish requires further investigations.

### 4.3 Migration trade‐offs

Upstream regions of the basin host the spawning sites used for migratory individuals, but they also harbor some resident fish, suggesting the reproduction may also occur without migration. However, many fish migrated, as indicated by the number of fishes leaving the spawning sites after the reproductive period. Given that migratory behaviour occurs through natural selection, the benefits of migration must outweigh those of residency (Sutherland, 1996). Higher weight and body condition factor at feeding sites suggest that the benefits of feeding migration outweigh the costs, likely due to the availability of food. Although detritus, the primary food resource for this species, is abundant and widely distributed in aquatic ecosystems (Araujo‐Lima et al., 1986; Bowen, 2022), the upper stretches used as spawning sites are situated in upstream areas where the rivers are smaller, narrower and have faster water velocity. In contrast, downstream stretches of the basin are wider, receive organic matter input from floodplains, and have lower water velocity, leading to increased detritus availability.

However, another possibility should be considered: there may be some form of balancing selection, whereby only certain individuals benefit from migrating, whereas others benefit more from remaining resident in spawning sites (apparently, the smaller ones). Under balancing selection, migrants and residents have, on average, equal fitness. Additionally, larger individuals can outcompete smaller ones when disputing the best feeding sites (lower stretches of the basin) in a territorial conflict, leaving upstream areas (coinciding with spawning grounds) for the smaller adults. Both possibilities represent interesting avenues for further investigation.

In any case, spawning is concentrated in the upstream areas of the basin, as fish reproductive behaviour may be related to indirect benefits to offspring by reproducing in sites that provide advantages during early development stages (Jørgensen et al., 2008). This pattern of spawning in upper reaches is well documented in Neotropical fishes (Pompeu et al., 2023), as it facilitates the downstream transport of free‐floating eggs. These eggs hatch while drifting, reaching floodplain lagoons as larvae, where the floodplain offers shelter and food for juvenile growth (Meschiatti et al., 2000), thereby enhancing survival.

### 4.4 Comprehensive new synthesis of the Pioa life cycle

Annually, adult pioa spend about 71% of the year in their feeding sites, migrating to spawning sites between late September and early October, often coinciding with rising river levels (Lopes et al., 2018). This migration, typically synchronized with the new and waxing moon phases, can extend until December (Lopes, Pompeu, et al. 2019). However, only a portion of individuals migrate each year and, in this study, we found that body weight, body condition factor, plasma 17β‐estradiol, testosterone, and cortisol are not related to migration decisions. We also observed resident fish at spawning sites, but these individuals were smaller and had lower body condition factors. In addition, some fish migrated two consecutive years, others did not migrate for 2 years, and some migrated only once within a 2‐year period. Migrating fish travel upstream at average speeds of 34.4 km/day, with no correlation to sex or biometric variables (Lopes, Alves, et al. 2019).

Spawning primarily occurs in the upper basin, particularly in the São Francisco River and its tributaries such as Bambuí and Samburá rivers (Lopes, Pompeu, et al. 2019, this study). Eggs and larvae were found simultaneously only in the Bambuí River near its confluence with the São Francisco River, suggesting that spawning may take place along an extended stretch of this river tributary. This interpretation is further supported by the observation that most fish entering the Bambuí River reached at least 50 km upstream from its confluence with the São Francisco River.

Eggs hatch while drifting, and the larvae continues moving downstream towards floodplains, which are recognized as mains nursery areas (Moreira, Peressin, Lopes, & Pompeu 2023). After spawning, and spending around 25% of their annual time at spawning sites, migrating adult fish return to their feeding sites between December and May, travelling at average speeds of 97.7 km/day, a journey that represents about 4% of their annual time (Lopes, Pompeu, et al. 2019; Lopes, Alves, et al. 2019).

Juveniles leave the floodplains for small rivers, where they reach near‐mature sizes (Moreira, Peressin, & Pompeu 2023). During recruitment, juveniles from growth sites undertake annual dispersal movements across the basin, locally known as ‘arribação’, to join the adult population (Prado et al., 2016). Upon reaching adulthood, fish exhibit partial migratory behaviour, showing fidelity to their spawning and feeding sites (Lopes et al., 2018, this study). These sites may vary in distance from a few to hundreds of kilometres apart, resulting in highly variable migration distances within the population. However, an individual's migration distance appears consistent.

### 4.5 Implications for conservation

Because the species relies on fixed spawning and feeding sites, any disruption between these two critical habitats directly affects population maintenance, once it is not clear whether individuals could locate new spawning and feeding sites if their original routes were blocked. Consequently, site fidelity may hinder or even prevent the use of alternative migratory routes when rivers are dammed. Blocking any route to a spawning site could result in significant population loss and reduced genetic diversity, even if other spawning remains accessible. For example, Ludwig et al. (2023) found that the species experienced significant genetic bottlenecks in the upper Sao Francisco River basin in recent decades, likely due to the construction of Três Marias dam in the 1960s, which restricted access to the region for individuals feeding in more distant areas.

Blockage of migratory routes can have several effects. Beyond preventing fish from accessing reproduction areas, the results of this study suggest that, even if some reproduction is maintained, fish isolated upstream by dams may experience size reduction, as demonstrated for Amazon catfishes (Hauser et al., 2024), consequently decreasing fishery yields and fecundity. Additionally, changes in hydrological conditions caused by dams, particularly lower temperatures and reduced dissolved oxygen in the water discharged, can decrease 17β‐estradiol levels, thereby inhibiting gonadal maturation downstream of dams (Arantes et al., 2010). Such alterations can also suppress reproductive migration, as observed in the study region, where fish consistently migrate through the main channel of the São Francisco River while avoiding the Pará River, which is regulated by upstream dams (Lopes et al., 2018; this study).

Moreover, this fidelity of species to both sites highlights the need for bidirectional connectivity, hindering the effectiveness of fishways as a management tool, because reservoirs can block both upstream and downstream movements of adults, and the drifting of eggs and larvae (Lopes et al., 2021; Pelicice et al., 2015). Therefore, we propose strategic changes in the environmental impact assessment process for hydropower licensing (Lopes et al., 2025). Specifically, when assessing the impacts of a new dam, particularly regarding the use of a fishway as a mitigation measure, several key factors must be thoroughly assessed, including the following: (i) Are the spawning sites located upstream of the dam preserved? (ii) Does the reservoir allow for the orientation and migration of adults upstream (Lopes et al., 2021)? (iii) Does the reservoir size and turbine operation allow for the downstream drift of eggs and larvae? (iv) Does the discharged water inhibit migration cues? (v) Does the reservoir size allow for the downstream migration of adults postspawning migration? (vi) Does the fishway enable bidirectional fish movement?

Answering these questions necessitates additional measures, such as determining the main spawning sites and migration routes of the population. We also recommend the reallocation of fishing law enforcement efforts, strengthening protection at spawning sites during the reproductive season, when fish populations are most vulnerable. Furthermore, we suggest shifting the fishing ban period to begin in early October to mid‐September, when most studied individuals initiate reproductive migration – rather than in November, as currently established. Finally, as our results further reveal, although the entire basin – including the main channel and major tributaries – functions as feeding sites for adults, spawning is exclusive to specific headwater areas. This underscores the critical importance of a limited number of spawning sites, which sustains the entire population by receiving fish from across the basin. As a conservation strategy, we recommend prioritizing enforcement efforts to prohibit fishing in these crucial areas during the closed season, reallocating resources from other regions if necessary. Notably, most spawning sites are located in small rivers, which often receive less public and regulatory attention.

### 4.6 Conclusions

The pioa exhibits migration patterns comparable to other phylogenetically distant fish groups, suggesting that our findings may be applicable to other Neotropical migratory species, and that shared management measures could be feasible. Our study provides a comprehensive synthesis of the pioa's life cycle, potentially making it one of the most detailed descriptions among Neotropical migratory fish. The insights gained are crucial for designing and implementing successful conservation programmes, including site conservation or restoration, connectivity enhancement, stock protection during reproduction or migration, and understanding the environmental cues driving migration. Given the pioa's widespread distribution across the basin, it can serve as an umbrella species, with conservation efforts benefiting numerous migratory and non‐migratory species. Additionally, migratory species can serve as sentinels of environmental change (Wikelski & Tertitski, 2016), with those failing to adapt phenologically being particularly vulnerable (Torstenson & Shaw, 2025). Therefore, it is imperative to maintain continuous annual migration records of Neotropical freshwater fish and expand studies to include other migratory species, a practice that is currently lacking for Neotropical migratory fish species.

## AUTHOR CONTRIBUTIONS

Alexandre Peressin: conceptualization, data curation, formal analysis, investigation, methodology, project administration, validation, visualization, writing – original draft preparation. João de Magalhães Lopes: conceptualization, funding acquisition, methodology, validation, writing – review and editing. Francisco Ricardo Andrade Neto: data curation, formal analysis, investigation, methodology, validation, visualization, writing – review and editing. Gilmara Junqueira Machado: data curation, investigation, methodology, validation. Carlos Bernardo Mascarenhas Alves: project administration, validation, writing – review and editing. Luis David Solis Murgas: conceptualization, methodology, resources, validation, writing – review and editing. Paulo Santos Pompeu: conceptualization, funding acquisition, methodology, project administration, resources, supervision, validation, writing – review and editing.

## FUNDING INFORMATION

This work was funded by Companhia Energética de Minas Gerais/Agência Nacional de Energia Elétrica Research and Development Program – P&D 612. Paulo S. Pompeu was awarded a research productivity grant (grant number: 302328/2022–0) by Conselho Nacional de Desenvolvimento Científico e Tecnológico (CNPq). Alexandre Peressin received a doctoral scholarship from Fundação de Amparo à Pesquisa de Minas Gerais (FAPEMIG n°: 11548/2018) and a postdoctoral scholarship from Fundação de Amparo à Pesquisa do Estado de São Paulo (FAPESP n°: 2023/17041–3).
